# Survey on the current status of rabies post-exposure prophylaxis clinics construction and the surveillance of rabies-exposed individuals in China, 2022

**DOI:** 10.1371/journal.pntd.0014011

**Published:** 2026-06-22

**Authors:** Qian Ren, Di Mu, Hong-Rui Zhai, Si-Han Li, Shi-Jian Zhou, Jun-Yuan Chen, Ao Luo, Yun-Long Zheng, Wen-Wu Yin, Yan-Ping Zhang, Qiu-Lan Chen

**Affiliations:** 1 National Key Laboratory of Intelligent Tracking and Forecasting for Infectious Diseases, Chinese Center for Disease Control and Prevention (Chinese Academy of Preventive Medicine), Beijing‌‌, China; 2 School of Public Health, Guangxi Medical University, Nanning‌‌, China; Colorado State University, UNITED STATES OF AMERICA

## Abstract

**Background:**

Rabies continues to pose a significant public health challenge in China. To offer a scientific foundation for formulating rabies prevention and control strategies, this study conducted a national survey to comprehend the current status of rabies post-exposure prophylaxis (PEP) clinics construction and the surveillance of rabies-exposed individuals in China.

**Methods:**

The Chinese Center for Disease Control and Prevention (CCDC) developed a national rabies prevention and control questionnaire, and conducted a survey across 31 provinces in Mainland China. The questionnaire was completed by the person in charge of rabies prevention and control in each provincial disease prevention and control institution.

**Results:**

By the end of 2022, 24,304 PEP clinics were reported in Mainland China. The number of PEP clinics per capita in China is 1.75 per 100,000. Nationwide, 47% of PEP clinics were capable of handling category III exposures. A total of 10,770,543 PEP clinic visits were reported. East China reported the highest number of rabies exposures (4,022,265). Among category III exposures, only 35% reported using rabies immunoglobulin (RIG). 97% of rabies exposures were attacked by dogs or cats. In South China, exposures caused by cats were nearly as frequent as those caused by dogs (47% and 48% respectively).

**Conclusions:**

The distribution of PEP clinics across China is uneven. This pattern appears broadly consistent with the historical geographic distribution of rabies risk, which may reflect a risk-informed allocation of resources. We recommended implementing tiered prevention and control strategies, with emphasis on enhancing the stockpile of RIG, dogs and cats population management, and health education in regions with higher rabies exposure risks. In provinces with a high incidence of rabies outbreaks, it is essential to actively advocate for the inclusion of rabies vaccines and RIG in the reimbursement scope of the New Rural Cooperative Medical Insurance Scheme, and raise the reimbursement rate and limit. The “One Health” approach should be adopted, including mandatory immunization and surveillance management systems for dogs and cats.

## Author summary

Rabies remains a significant public health issue in China despite considerable progress in reducing human cases. This nationwide survey assessed the status of rabies post-exposure prophylaxis (PEP) clinics construction and the surveillance of rabies-exposed individuals in 2022. We found that while China has established over 24,000 PEP clinics, their distribution is uneven across regions, with higher densities in central provinces. Only 47% of clinics are equipped to handle the most severe exposures (category III), and among these, merely 35% of rabies-exposed individuals received rabies immunoglobulin (RIG). Most exposures were caused by dogs and cats, with regional variations in age group affected. Our findings highlight the need for a tiered prevention strategy, better RIG availability, stronger dog and cat vaccination programs, and the inclusion of rabies vaccines and RIG in medical insurance schemes in high-risk regions. Adopting a “One Health” approach is essential to achieving the goal of eliminating dog-mediated human rabies by 2030.

## Introduction

Rabies is an acute zoonotic infectious disease caused by the infection of the rabies virus. Its clinical manifestations are characteristic, including symptoms such as fear of wind, fear of water, pharyngeal muscle spasms, and progressive paralysis [1]. Although the incidence rate of rabies is low, the case fatality rate is nearly 100% once it occurs [2–4]. According to recent surveys, rabies causes approximately 59,000 deaths globally each year and has become a significant public health problem, especially in Asian and African countries [5,6]. Rabies imposes a heavy economic burden on individuals, families, and society at large. Globally, it is estimated that rabies causes annual economic losses reaching as high as $8.6 billion [7]. Post-exposure prophylaxis (PEP) is a key measure for controlling human rabies outbreaks. Timely and standardized PEP can prevent almost all cases [8].

From 1950 to 2022, China reported a total of 132,847 cases of rabies. During this period, China experienced three peaks of rabies outbreaks, which occurred in 1956 (1,942 cases), 1981 (7,037 cases), and 2007 (3,300 cases) [9]. From 2007 to 2022, the national rabies epidemic showed a continuous downward trend over 16 years. In 2011, the number of reported cases was fewer than 2,000; in 2014, it was fewer than 1,000; and by 2022, only 133 cases were reported. Compared with the peak of 3,300 cases in 2007, the number of cases in 2022 decreased by 96%. Rabies occurs throughout the year, with a relatively higher incidence in spring, summer, and autumn, and a lower incidence in winter [10]. All age groups are at risk of contracting the disease, and the differences in incidence among different genders, ages, and occupational groups are related to the opportunity to come into contact with dogs and other animals [11].

In 2015, the World Health Organization (WHO), the World Organization for Animal Health (WOAH), the Food and Agriculture Organization of the United Nations (FAO), and the Global Alliance for Rabies Control (GAARC) jointly set the goal of eliminating dog-mediated human rabies by 2030, which implies achieving zero deaths from dog-mediated human rabies globally [12–14]. In response to the requirements of the WHO to enhance surveillance of rabies-exposed individuals, to gain a better understanding of the actual status of rabies prevention and control efforts across China, and to provide a basis for formulating scientific and reasonable rabies prevention and control strategies, this study designed a descriptive, nationwide cross-sectional survey to map the current status of rabies PEP clinics distribution and the surveillance of rabies-exposed individuals nationwide.

## Methods

### Ethics statement‌‌

Data for this study were derived from continual public health monitoring of a designated notifiable infectious disease. The National Health and Family Planning Commission of China classified the investigation of human rabies incidents as a perpetual public health surveillance effort, exempting it from institutional ethical review board evaluation. Similarly, this project was recognized as a standard public health surveillance activity, aligning with the human subjects’ protection protocols of the United States CDC (CGH #2015–238). All data used in the analysis were anonymized, containing no personally identifiable information. All investigations were conducted with verbal informed consent from the patient’s family.

1. Study design: A descriptive, nationwide cross-sectional survey was conducted using a standardized questionnaire developed by the Chinese Center for Disease Control and Prevention (CCDC). As this was a census-style survey covering all 31 provinces in mainland China, no sampling was performed. The primary analysis aimed to present national and regional summaries of PEP clinics coverage and exposure characteristics. Secondary analyses involved comparing the proportional distribution of exposure categories, age groups, and animal types across geographical regions using chi-square tests to identify significant variations.
2. Survey subjects: The survey encompassed 31 provinces (autonomous regions and municipalities directly under the Central Government) in Mainland China, excluding the Hong Kong Special Administrative Region, the Macao Special Administrative Region, and Taiwan, China. The questionnaire was completed by the person in charge or key personnel responsible for rabies prevention and control in each provincial disease prevention and control institution.
3. Survey content: The survey focused on the construction status of PEP clinics and the surveillance of rabies-exposed individuals in 2022. This included the number of PEP clinics, their capacity to handle different exposure categories, as well as the number of exposures, exposure category, age distribution, and types of animals involved.
4. Exposure category: Rabies exposure refers to being bitten, scratched, or having mucous membranes or broken skin licked by a rabid, suspected rabid, or undetermined animal host. It also includes direct contact of open wounds or mucous membranes with saliva or tissue potentially containing the rabies virus [15].
5. Based on the type of contact and severity of exposure, rabies exposure is classified into three levels [15–17]. Category I exposure involves touching or feeding an animal, or having intact skin licked. Category II exposure occurs when uncovered skin is lightly bitten, or when minor scratches or abrasions without obvious bleeding are sustained. Category III exposure includes single or multiple penetrating bites or scratches to the skin, licking of broken skin, contamination of open wounds or mucous membranes with saliva or tissue, or direct contact with bats.
6. Quality control: A database was constructed using Epidata 3.1 software. Double entry of questionnaire data, logical error checking, and data cleaning on the questionnaire data were carried out. Missing information was supplemented through telephone follow-up, etc.
7. Rabies case data: The rabies case data were obtained from the China Information System for Disease Control and Prevention (CISDCP).
8. Statistical analysis: Descriptive analysis of the data was performed using Excel 2016 software. Since our data are aggregated at the provincial level, chi-square tests were conducted using R 4.4.2 to compare characteristics of exposed individuals across regions.

## Results

### Current situation of PEP clinics construction

PEP clinics density varied widely across China. By the end of 2022, a total of 24,304 PEP clinics were reported in 30 provinces in Mainland China, excluding Jilin Province. The number of rabies PEP clinics in East China exceeded 7,000, which was significantly higher than that in other regions. The number of rabies PEP clinics in Hunan and Shandong provinces exceeded 2,000. Twenty-seven provinces have established PEP clinics in all districts (counties and county-level cities). Nationwide, 47% of PEP clinics can handle category III exposures. PEP clinics in Shandong, Hubei, Sichuan, Beijing, and Shanghai are all capable of handling category III exposures.

**Fig 1:**
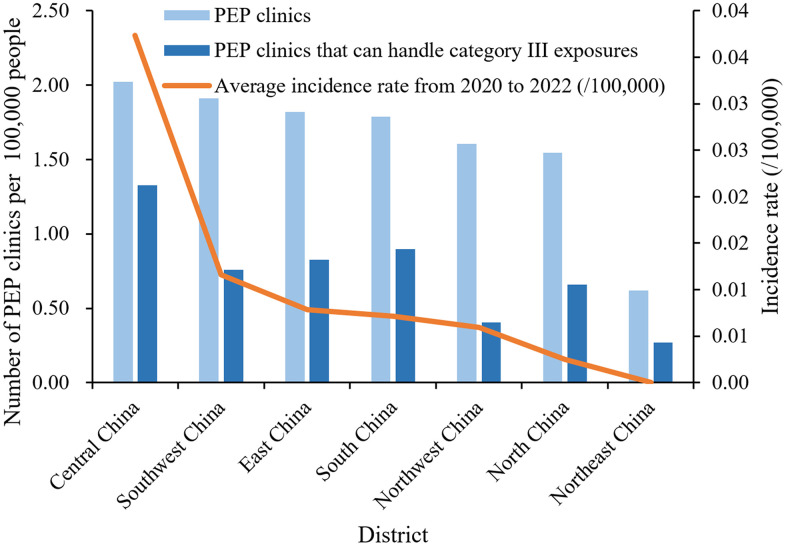
Current situation of PEP clinics construction and average incidence rate from 2020 to 2022.

China had 1.75 PEP clinics per 100,000 people, with central China having the highest density at 2.02 per 100,000 people. Northeast China had only 0.62 clinics per 100,000, which is 35% of the national average. The number of clinics capable of handling category III exposures was 0.82 per 100,000 nationally, with Central China having the highest (1.33) and Northeast China the lowest (0.27, 33% of the national average). The average annual incidence of rabies from 2020 to 2022 was 0.012 per 100,000 nationally, highest in Central China (0.037), with no cases reported in the Northeast (Fig 1).

In 2022, 21 provinces in China established PEP outpatient information management systems to monitor vaccination status (17 provinces), dog-induced injuries (13 provinces), immunization history of animals causing injuries (8 provinces), and multiple injuries caused by a single dog (6 provinces). Meanwhile, 19 provinces have formulated relevant standards for the standardized construction of PEP clinics. Moreover, provinces like Guangdong, Zhejiang, Shaanxi, and Hunan have incorporated PEP expenses into their medical insurance.

### Basic information on the exposed population

#### Number of exposures and exposure category distribution.

**Fig 2:**
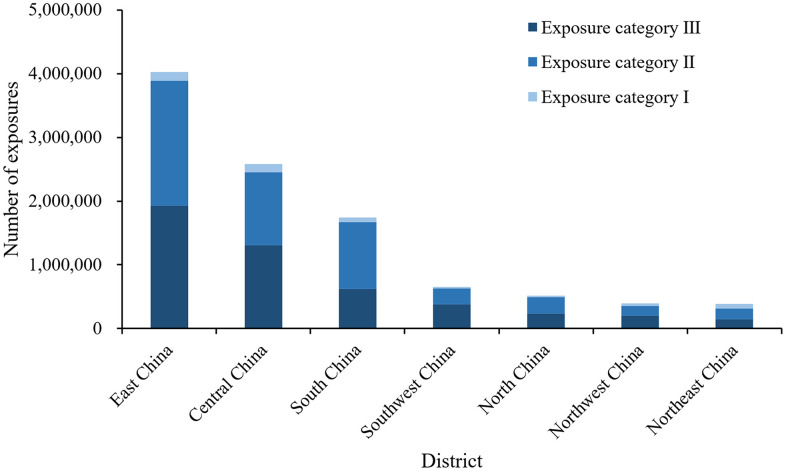
Number of exposures and exposure category distribution.

In 2022, apart from Qinghai, Jilin, Beijing, Yunnan, and Sichuan, 26 provinces in Mainland China reported a total of 10,770,543 visits to PEP clinics. The male-to-female ratio was approximately 1:1. East China reported the highest number of visits (4,022,265), followed by Central China and South China. Southwest China, North China, Northwest China, and Northeast China reported fewer exposures. The distribution of exposure categories varied significantly across regions (*P* < 0.05), with Southwest China having the highest proportion of category III exposures (58%) (Fig 2). This notably high proportion suggests a potentially higher risk of animal injuries, more conservative clinical judgment by physicians, or lower public awareness of protection in this region. Only 35% of category III exposures received RIG.

### Age distribution of exposed individuals

**Fig 3:**
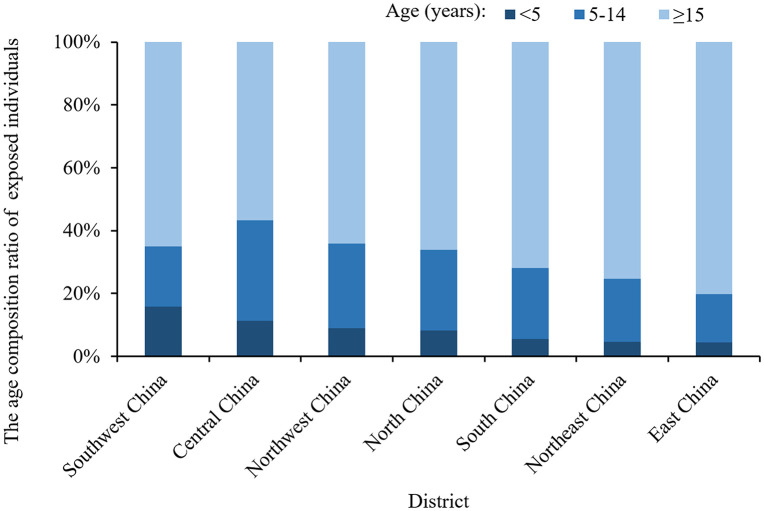
Age distribution of exposed individuals.

The age distribution of exposed individuals varied significantly across regions (*P* < 0.05), with notable clustering of young children in Southwest China and older children/adolescents in Central China. Southwest China had the highest proportion of children under 5 years old (16%), while Central China had the highest proportion of children or adolescents aged 5–14 (32%) (Fig 3).

### Attacking animals’ distribution of exposed individuals

**Fig 4:**
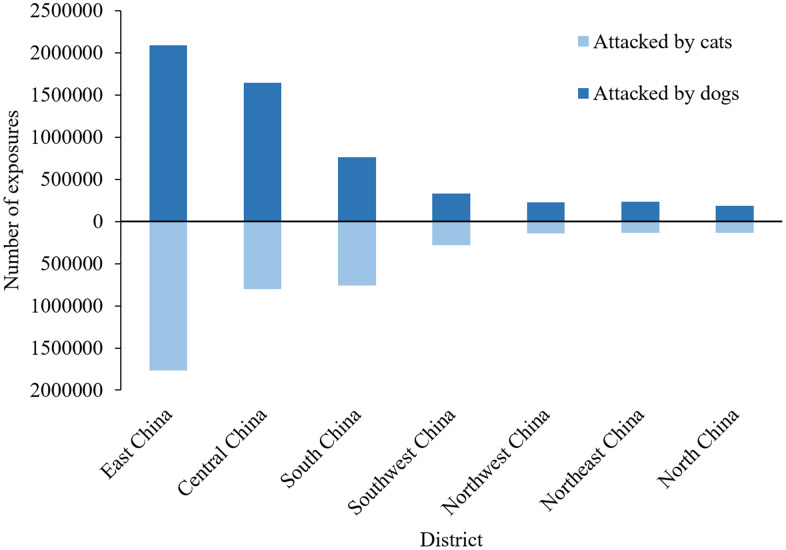
Distribution of dog and cat attacks on exposed individuals.

Among exposures where the attacking animal was reported, dogs and cats together accounted for 97% of cases. The relative proportion of injuries caused by cats (versus dogs) varied significantly by region (*P* < 0.05). In South China, exposures caused by cats were nearly as frequent as those caused by dogs (47% and 48% respectively). (Fig 4).

## Discussion

Over the past decade, China has achieved remarkable progress in the prevention and control of rabies [7,18]. From 2007 to 2022, owing to the robust prevention and control measures implemented by the Chinese government, the national rabies epidemic has exhibited a downward trend for 16 consecutive years. Currently, rabies is in a sporadic state across the nation, laying the foundation for the elimination of dog-mediated human rabies [19–21].

PEP is a key measure for controlling human rabies outbreaks [6,8,15]. Extensive publicity and education regarding PEP should be conducted to enhance public awareness of protection and encourage proper vaccination against rabies. In 2022, a total of 24,304 PEP clinics were reported across the nation. Twenty-seven provinces have established PEP clinics in all districts (counties and county-level cities) under their jurisdiction, achieving near complete coverage at the county level.

Currently, China has not established a unified standard for the number of PEP clinics per capita. This cross-sectional study shows that the number of PEP clinics per capita in China is 1.75 per 100,000 people. Central China has the highest density (2.02 per 100,000), while Northeast China (0.62 per 100,000) has only 35% of the national average. Although Northeast China has the fewest PEP clinics per capita, no rabies cases were reported there from 2020 to 2022. These findings suggest that despite regional disparities in the distribution of PEP clinics due to factors such as economic development, the pattern appears broadly consistent with the historical geographic distribution of rabies risk, which may reflect a risk-informed allocation of resources.

This study indicates that East China, Central China, and South China reported the highest number of rabies exposures. It is recommended to implement a tiered prevention and control strategy, focusing on enhancing the stockpile of RIG, dog population management, and conducting multi-dimensional health education in these high-risk regions. Operationalizing this tiered strategy requires a clear, data-driven framework. We propose that the national rabies control program formally adopts a risk-classification system for counties, integrating indicators such as historical human incidence, dog vaccination coverage, and PEP clinics accessibility. High-risk counties could then be mandated to implement enhanced measures, including quarterly monitoring of RIG stockouts, intensive dog vaccination drives, and dedicated funding for community engagement. Such a system would ensure that resources and interventions are precisely targeted where they are most needed.

In this survey, the proportion of category III exposures in Southwest China was as high as 58%, reflecting either a higher risk of animal injuries, more conservative clinical judgment by physicians, or lower public awareness of protection in this region. According to the Regulations for the Prevention and Disposal of Rabies Exposure (2023 edition), cases with category III exposure should be administered RIG [15,17]. However, in China, the utilization of RIG for category III exposures is relatively low [6]. In this survey, only 35% of category III exposures received RIG. The cost of PEP for rabies vaccination alone is approximately 300 yuan per individual, and if RIG is needed, the cost surpasses 1,000 yuan [6]. Additionally, it also involves expenses such as wound treatment and work absence. Over 70% of rabies cases in China occur in rural regions. In 2020, the per capita disposable income of the rural population in China was 17,131 yuan, imposing a substantial economic burden on low-income populations [7]. Therefore, it is imperative to actively encourage provinces with a high incidence of rabies outbreaks to incorporate rabies vaccines and RIG into the reimbursement scope of the New Rural Cooperative Medical Insurance Scheme and raise the reimbursement rate and limit. To translate this finding into actionable steps, we recommend initiating pilot programs in high-burden provinces that combine targeted cost subsidies or full insurance coverage for RIG to directly alleviate the financial barrier, and standardized training and decision-support tools for clinicians at PEP clinics to ensure consistent application of WHO guidelines for RIG administration. Evaluating the impact of such pilots on RIG utilization and ultimately on rabies case reduction would provide critical evidence for national policy scaling.

In Southwest China, children under 5 years old accounted for 16% of exposures, while in Central China, children or adolescents aged 5–14 accounted for 32%. These findings highlight the need for targeted health education programs focusing on improving risk awareness among guardians of children and self-protection skills among adolescents. Specifically, public health campaigns in Southwest China could prioritize community-based interventions for parents and kindergarten staff, focusing on supervision during child-animal interactions. In Central China, school-based educational programs tailored for primary and middle school students, incorporating interactive materials on safe behavior around animals and post-exposure first aid, should be developed and their effectiveness assessed. Collaboration with education departments would be essential for implementing and sustaining these interventions.

Analysis of animal attacking exposure shows that dogs remain the primary source of transmission route, responsible for 56% of exposures, which is consistent with previous reports [12]. Based on global experience in eliminating dog-mediated rabies, continuous vaccination of dogs with an immunization coverage rate exceeding 70% for at least 3–7 years can block dog-mediated rabies. This method is cost-effective and acts as the first line of defense against human rabies [6,8,22]. Therefore, it is necessary to explore sustainable multi-sectoral joint prevention and control mechanisms, implement the concept of “One Health”, strengthen dog management, improve dog immunization coverage, and eliminate dog-mediated rabies [16,23–25].

In this survey, 41% of patients were injured by cats. With the improvement of living conditions and the ecological environment in China, the number of pets, such as cats, is increasing. As pet ownership grows, it is imperative to implement the “One Health” concept and establish comprehensive immunization, surveillance, and management systems for companion animals. At the same time, the population density of wild carnivores is also increasing annually [26–29]. The risk of rabies associated with these animals is becoming increasingly prominent.

This study has several limitations. First, as a cross-sectional survey based on provincial-level aggregated data, it does not capture individual-level exposure patterns, treatment adherence, or clinical outcomes. Crucially, the use of aggregated data precludes causal inference and limits the depth of our analysis regarding the effectiveness or optimization of PEP clinics allocation. Second, despite efforts to supplement missing information through telephone follow-up and other methods, some data remained unavailable, which may affect the completeness of the national picture. Future studies incorporating patient-level records, longitudinal designs, and mixed-methods approaches are needed to validate these findings and provide stronger evidence for guiding rabies elimination strategies. However, we believe that this study can still indicate the current status of rabies prevention and control in China and provide a scientific basis for formulating rabies prevention and control strategies.

## Conclusions

This nationwide survey provides a systematic overview of the PEP clinics infrastructure and rabies exposure surveillance in China in 2022. The key findings reveal an uneven geographical distribution of clinics, with inadequate capacity to manage severe (category III) exposures in nearly half of all clinics and in certain regions. Suboptimal utilization of RIG was identified as a critical gap in clinical management. Furthermore, the epidemiology of exposures showed distinct regional patterns, including a high burden among children and adolescents in specific areas and a notable proportion of injuries caused by cats, particularly in South China. These findings highlight several priority areas for the national rabies elimination program. Future efforts should focus on strengthening PEP clinics capacity in underserved regions, investigating and addressing the barriers to RIG use, and implementing targeted prevention programs for high-risk groups and locations. Ultimately, sustaining progress toward the 2030 elimination goal will require enhanced “One Health” coordination to improve dog and cat vaccination coverage, alongside continued refinement of human surveillance systems based on robust, localized data.

## Abbreviations

PEP: post-exposure prophylaxis
CCDC: Chinese Center for Disease Control and Prevention
RIG: rabies immunoglobulin
WHO: World Health Organization
WOAH: World Organization for Animal Health
FAO: Food and Agriculture Organization of the United Nations
GAARC: Global Alliance for Rabies Control
CISDCP: China Information System for Disease Control and Prevention

## References

[1] Hemachudha T, Laothamatas J, Rupprecht CE. Human rabies: a disease of complex neuropathogenetic mechanisms and diagnostic challenges. Lancet Neurol. 2002;1(2):101–9. doi: 10.1016/s1474-4422(02)00041-8 12849514

[2] Jin J. Rabies. JAMA. 2023;329(4):350. 36692559 10.1001/jama.2022.22254

[3] Yin C. Progress in the Development of Animal Rabies Vaccines in China. China CDC Wkly. 2021;3(39):825–30. doi: 10.46234/ccdcw2021.204 34595001 PMC8477054

[4] Peterson D, Barbeau B. Human rabies - Utah, 2018. MMWR Morb Mortal Wkly Rep. 2020;69(5):121–4. 32027626 10.15585/mmwr.mm6905a1 PMC7004398

[5] Hampson K, Coudeville L, Lembo T, Sambo M, Kieffer A, Attlan M, et al. Estimating the global burden of endemic canine rabies. PLoS Negl Trop Dis. 2015;9(4):e0003709. doi: 10.1371/journal.pntd.0003709 25881058 PMC4400070

[6] Guo C, Li Y, Huai Y, Rao CY, Lai S, Mu D, et al. Exposure history, post-exposure prophylaxis use, and clinical characteristics of human rabies cases in China, 2006-2012. Sci Rep. 2018;8(1):17188. doi: 10.1038/s41598-018-35158-0 30464190 PMC6249250

[7] Chen Q. Accelerate the Progress Towards Elimination of Dog-Mediated Rabies in China. China CDC Wkly. 2021;3(39):813–4. doi: 10.46234/ccdcw2021.200 34594997 PMC8477055

[8] WHO. WHO Expert Consultation on Rabies. Geneva: World Health Organization. 2018.

[9] Tao X, Liu S, Zhu W, Rayner S. Rabies surveillance and control in China over the last twenty years. Biosafety and Health. 2021;3(3):142–7. doi: 10.1016/j.bsheal.2020.11.004

[10] Yin WW, Wang CL, Chen QL, Dong GM, Li YH, Zhu WY, et al. Expert consensus on rabies exposure prophylaxis. Zhonghua Yu Fang Yi Xue Za Zhi. 2019;53(7):668–79. doi: 10.3760/cma.j.issn.0253-9624.2019.07.004 31288336

[11] Qin Y, Zhang Q. Analysis of epidemic characteristics of human rabies in China in 2007-2023. Chinese Journal of Experimental and Clinical Virology. 2024;38(4):373–7.

[12] Chen Q, Ma X, Rainey JJ, Li Y, Mu D, Tao X, et al. Findings from the initial Stepwise Approach to Rabies Elimination (SARE) Assessment in China, 2019. PLoS Negl Trop Dis. 2021;15(3):e0009274. doi: 10.1371/journal.pntd.0009274 33780454 PMC8006992

[13] Abela-Ridder B, Knopf L. The beginning of the end of rabies?. Lancet Glob Health. 2016;4(11):e780–1. 10.1016/S2214-109X(16)30245-5 27692777

[14] Tidman R, Thumbi SM. United Against Rabies Forum: The One Health Concept at Work. Front Public Health. 2022;10:854419. 35493394 10.3389/fpubh.2022.854419 PMC9043483

[15] Rabies Exposure Prevention and Disposal Practice. 2023. https://wsjk.sjz.gov.cn/col/1585276611735/2023/09/26/1695716989967.html

[16] Chen Q, Liu Q, Gong C, Yin W, Mu D, Li Y, et al. Strategies to inTerrupt RAbies Transmission for the Elimination Goal by 2030 In China (STRATEGIC): a modelling study. BMC Med. 2023;21(1):100. doi: 10.1186/s12916-023-02821-x 36927437 PMC10022085

[17] World Health O. Rabies vaccines: WHO position paper, April 2018 - recommendations. Vaccine. 2018;36(37):5500–3. 30107991 10.1016/j.vaccine.2018.06.061

[18] Li H, Liu J-J, Ding S-J, Cai L, Feng Y, Yu P-C, et al. Human rabies in China: evidence-based suggestions for improved case detection and data gathering. Infect Dis Poverty. 2020;9(1):60. doi: 10.1186/s40249-020-00672-9 32487256 PMC7266119

[19] Shen T, Welburn SC, Sun L, Yang G-J. Progress towards dog-mediated rabies elimination in PR China: a scoping review. Infect Dis Poverty. 2023;12(1):30. doi: 10.1186/s40249-023-01082-3 37024944 PMC10077633

[20] Sharan M, Vijay D, Yadav JP, Bedi JS, Dhaka P. Surveillance and response strategies for zoonotic diseases: a comprehensive review. Sci One Health. 2023;2:100050. doi: 10.1016/j.soh.2023.100050 39077041 PMC11262259

[21] Zhang Q, Liu J, Han L, Li X, Zhang C, Guo Z, et al. How far has the globe gone in achieving One Health? Current evidence and policy implications based on global One Health index. Sci One Health. 2024;3:100064. doi: 10.1016/j.soh.2024.100064 39077388 PMC11262257

[22] Liu Z, Liu M. *Epidemic Characteristics of Human Rabies - China, 2016-2020.* China CDC Wkly, 2021. 3(39):819–21. 10.46234/ccdcw2021.203 PMC8477051 34594999

[23] Yin W, Fu ZF, Gao GF. Progress and Prospects of Dog-Mediated Rabies Elimination in China. China CDC Wkly. 2021;3(39):831–4. doi: 10.46234/ccdcw2021.205 34595002 PMC8477050

[24] Feng Y, Ma J. Epidemiology of Animal Rabies - China, 2010-2020. China CDC Wkly. 2021;3(39):815–8. 34594998 10.46234/ccdcw2021.202 PMC8477053

[25] Miao F, Li N, Yang J, Chen T, Liu Y, Zhang S, et al. Neglected challenges in the control of animal rabies in China. One Health. 2021;12:100212. doi: 10.1016/j.onehlt.2021.100212 33553562 PMC7843516

[26] Fehlner-Gardiner C, Gongal G. Rabies in Cats-An Emerging Public Health Issue. Viruses. 2024;16(10). 10.3390/v16101635 PMC11512395 39459967

[27] Meriño-Olivella S, Del Pilar Sánchez-Bonilla M, Escobar LE, Correa-Valencia NM. Human cat borne rabies as the new epidemiology of the disease in the Andes mountains. Zoonoses Public Health. 2024;71(5):600–8. doi: 10.1111/zph.13141 38706119

[28] Ma X, Boutelle C, Bonaparte S, Orciari LA, Condori RE, Kirby JD, et al. Rabies surveillance in the United States during 2022. J Am Vet Med Assoc. 2024;262(11):1518–25. doi: 10.2460/javma.24.05.0354 39059444

[29] Feng Y, Wang Y. Diversity of rabies virus detected in Inner Mongolia, China, 2019-2021. Transbound Emerg Dis. 2022;69(2):249–53. 35001535 10.1111/tbed.14451

